# Cardiac magnetic resonance in patients with ARVC and family members: the potential role of native T1 mapping

**DOI:** 10.1007/s10554-021-02166-7

**Published:** 2021-02-07

**Authors:** Georgios Georgiopoulos, Mattia Zampieri, Silvia Molaro, Anna Chaloupka, Alberto Aimo, Barbara Barra, Leema Roberts, Laura Monje-Garcia, Colin Evans, Nabeel Sheikh, Rachel Bastiaenen, Michael Cooklin, Pier-Giorgio Masci, Gerald Carr-White, Gherardo Finocchiaro, Amedeo Chiribiri

**Affiliations:** 1 Department of Cardiovascular Imaging, School of Biomedical Engineering and Imaging Sciences, Guy’s and St Thomas’ NHS Foundation Trust, London, UK; 2 Inherited Cardiac Conditions Service, Guy’s and St Thomas’ NHS Foundation Trust, London, UK; 3 Institute of Life Sciences, Scuola Superiore Sant’Anna, Pisa, Italy; 4 Cardiology Division, University Hospital of Pisa, Pisa, Italy; 5 Wellcome Trust Medical Engineering Centre, King’s College London, London, UK; 6 Cardiology Division, University Hospital of Pisa, and Institute of Life Sciences, Scuola Superiore Sant’Anna, Piazza Martiri della Libertà 33, Pisa, Italy

**Keywords:** ARVC, T1 mapping, Late gadolinium enhancement, Diagnosis

## Abstract

Left ventricular (LV) involvement in patients with arrhythmogenic right ventricular cardiomyopathy (ARVC) is not evaluated in the revised Task Force Criteria, possibly leading to underdiagnosis. This study explored the diagnostic role of myocardial native T1 mapping in patients with ARVC and their first-degree relatives. Thirty ARVC patients (47% males, mean age 45 ± 27 years) and 59 first-degree relatives not meeting diagnostic criteria underwent CMR with native T1 mapping. C MR was abnormal in 26 (87%) patients with ARVC. The right ventricle was affected in isolation in 13 (43%) patients. Prior to T1 mapping assessment, 2 (7%) patients exhibited isolated LV involvement and 11 (36%) patients showed features of biventricular disease. Left ventricular involvement was manifest as detectable LV late gadolinium enhancement (LGE) in 12 out of 13 cases. According to pre-specified inter-ventricular septal (IVS) T1 mapping thresholds, 11 (37%) patients revealed raised native T1 values including 5 out of the 17 patients who would otherwise have been classified as exhibiting a normal LV by conventional imaging parameters. Native septal T1 values were elevated in 22 (37%) of the 59 first-degree relatives included. Biventricular involvement is commonly observed in ARVC; native myocardial T1 values are raised in more than one third of patients, including a significant proportion of cases that would have been otherwise classified as exhibiting a normal LV using conventional CMR techniques. The significance of abnormal T1 values in first-degree relatives at risk will need validation through longitudinal studies.

**Supplementary Information:**

The online version of this article (10.1007/s10554-021-02166-7) contains supplementary material, which is available to authorized users.

## Background

Arrhythmogenic right ventricular cardiomyopathy (ARVC) is an inherited heart muscle disease characterized by progressive replacement of the ventricular myocardium by fibro-fatty tissue predisposing to life-threatening arrhythmias [1]. The diagnosis of ARVC is often complex and based on the revised Task Force Criteria (TFC) which require the presence of several clinical, structural, electrocardiographic, and histopathological changes [2]. The current TFC fail to address the issue of left ventricular (LV) involvement in the disease process, which is increasingly recognized and observed in up to 80% of relatives of sudden death victims diagnosed with ARVC on post-mortem examination [3]. The recently proposed “Padua criteria” give the proper importance to tissue characterization by cardiovascular magnetic resonance (CMR), in the setting of a multidimensional evaluation [4] (Supplemental Table 1).

CMR plays a central role in the diagnosis of ARVC because of its ability to accurately assess regional wall motion abnormalities, chamber volumes and systolic function. Late gadolinium enhancement (LGE) at CMR can be a sign of focal myocardial fibrosis, but subtle or diffuse fibrotic changes may be missed. Conversely, T1 mapping enables the detection of an increase in extracellular volume and diffuse myocardial fibrosis and has proved useful in differentiating between different cardiomyopathy subtypes characterized by left ventricular hypertrophy [5, 6]. To date, there is limited data on the role of T1 mapping in ARVC [7].

The aim of this study was to explore a possible diagnostic role of pre-contrast or native myocardial T1 mapping in patients with ARVC and in first-degree relatives, and to investigate the relationship between LV involvement on CMR and 12-lead electrocardiogram (ECG) abnormalities.

## Methods

### Study population

#### Patients with arrhythmogenic right ventricular cardiomyopathy

Between 2012 and May 2019, 73 patients with ARVC according to TFC underwent CMR examination at the King’s College London Department of Cardiovascular Imaging as part of their assessment in the inherited cardiac conditions (ICC) clinic at Guy’s and St Thomas’ Hospital. All patients underwent comprehensive evaluation including personal and family history, clinical examination, 12-lead ECG, signal-averaged ECG, transthoracic echocardiogram, exercise tolerance test and 24-h ECG Holter monitoring. Clinical data were retrospectively evaluated, and the final study population consisted of 30 patients with ARVC in whom pre-contrast T1 mapping sequences had been acquired with image quality adequate for analysis. T1 mapping at our institution has been used consistently for patients who consented for their imaging data to be used for research purpose from early 2017; therefore, the majority of patients included in the final study cohort had been investigated between 2017 and 2019. Genetic testing was performed in 18/30 (60%) patients.

#### First‐degree relatives

During the same time-period, first-degree relatives of patients with ARVC were offered comprehensive diagnostic work-up, including a 12-lead ECG, transthoracic echocardiogram, exercise tolerance test, 24-h Holter monitor and CMR. A total of 130 first-degree relatives were investigated. The current study comprised 59 first-degree relatives in whom T1 mapping sequences were acquired with image quality adequate for analysis. Genetic testing was performed in 15 individuals.

The study was approved by the Institutional Ethics Committee and all patients with ARVC and first-degree relatives provided written informed consent prior to screening for the CMR images and related clinical data to be anonymously analyzed for research. Overall, the study was conducted in full compliance with the principles of Good Clinical Practice and the Declaration of Helsinki [8].

### 12-Lead electrocardiogram

Standard 12-lead ECGs were performed as described elsewhere [9]. Care was taken when measuring the extent of T-wave inversion (TWI) across the precordial leads and the maximum J-point elevation in the anterior leads (V1–V4) exhibiting TWI. The amplitude of the J-point was measured at the end of the QRS complex (the onset of the ST-segment) with reference to the onset of the QRS complex [10, 11]. Sokolow–Lyon voltage criterion for LV hypertrophy was defined as the sum of the S-wave in V_1_ and the R-wave in V_5_ or V_6_ (whichever was larger in amplitude) being ≥ 0.35 mV. The J-point amplitude was measured at the end of the QRS complex (the onset of the ST-segment) with reference to the onset of the QRS complex [12] and was considered elevated if ≥ 0.1 mV. The S-wave duration in leads V1–V3 was considered prolonged if > 55 ms. ST-segment depression was considered significant if ≥ − 0.1 mV in ≥ 2 contiguous leads. Biphasic T-wave inversion was considered abnormal if the negative deflection of the T-wave exceeded ≥ − 0.1 mV. T-wave inversion ≥ 0.1 mV in ≥ 2 contiguous leads was considered abnormal. Deep T-wave inversion was defined as a T-wave deflection ≥ − 0.2 mV. An abnormal Q-wave was defined as a Q-wave with duration ≥ 40 ms or a Q/R ratio > 0.25. The normal frontal cardiac axis was defined as > − 30° and < 120°. Left atrial (LA) enlargement was defined by a P-wave duration ≥ 0.12 s in the frontal plane associated with a terminal P negativity in lead V1 of duration ≥ 40 ms and depth ≥ 0.1 mV. Low ECG voltages were defined as QRS amplitude ≤ 1.0 mV in all of the precordial leads and/or QRS amplitude ≤ 0.5 mV in all of the limb leads [13]. T-wave inversion in V1–V3 was considered a normal juvenile ECG pattern in asymptomatic patients < 16 years old [14].

### Cardiovascular magnetic resonance imaging

Cardiovascular magnetic resonance studies were performed using 1.5T or 3T scanners (Achieva or Ingenia, Philips Healthcare; Aera, Siemens), using steady-state free precession (SSFP) breath-hold cines in long-axis planes and sequential 7 mm short-axis slices from the atrioventricular ring to the apex [15]. Ventricular volumes and function and LV mass were measured using standard techniques [16]. Ventricular volumes and LV mass were indexed for age and body surface area, BSA [17]. Right ventricular regional wall motion abnormalities (RWMA) were classified as akinesia, dyskinesia and aneurysms [2]. Late gadolinium enhancement images were acquired 10 min after an intravenous bolus injection of 0.1 mmol/kg gadoterate meglumine (Dotarem) or 0.15 mmol/kg of Gadovist to identify regional fibrosis. Inversion times were adjusted to null normal myocardium and LGE images were phase swapped to exclude artifact when required. We considered the CMR RV volume and ejection fraction threshold values proposed by the revised TFC as diagnostic for ARVC [2] (in combination with RV RWMAs where relevant).

#### T1 mapping

In 30 patients with ARVC and 59 first degree relatives who consented for research, balanced SSFP single breath-hold modified inversion recovery Look-Locker (MOLLI) sequences were used for T1 mapping in a single mid-ventricular short axis slice, prior to contrast administration.

Among patients with ARVC, 18 were scanned on a 1.5T scanner and 12 on a 3T scanner (Supplemental Table 2). Among first-degree relatives, 42 were scanned on a 1.5T scanner and 17 on a 3T scanner. Native T1 mapping was implemented according to the consensus statement by the Society for Cardiovascular Magnetic Resonance (SCMR) 2017, using cvi42 software (Circle Cardiovascular Imaging version 5.6.6, Calgary, Canada) 5. Myocardial T1 mapping values were measured by placing a standardized region of interest (ROI) in the short axis slice within the mid inter-ventricular septum and in the mid lateral wall. Care was taken to avoid contamination with signal from the blood pool and areas of LGE. T1 mapping was only performed for the LV alone as the thin right ventricle (RV) wall renders T1 mapping susceptible to partial volume effects.

We used previously published reference values for native septal T1 values in healthy volunteers according to used scanner (1.5 or 3T) [18] and defined abnormally increased measurements as values exceeding mean ± 3SD (i.e. > 99th percentile of the normal distribution for native pre-contrast T1 values, respectively). Given the absence of reference values for the lateral wall in segmental T1 mapping, we did not adjudicate on native T1 values of the mid lateral segments [18–20]. Of interest, the T1-mapping sequence and imaging protocol for derivation of reference values has been standardized and validated at our CMR department at King’s College London [18]. Two experienced researchers in CMR analyzed native T1 myocardial values blinded to patients’ status (ARVC or 1st degree relatives).

### Statistical analysis

Results are expressed as mean ± SD for continuous variables and as absolute numbers and relative percentages for categorical variables. Comparison between groups was performed using Student’s t-test for independent samples or the non-parametric Kruskal–Wallis test for continuous outcomes, and the chi-squared test or Fisher’s exact test for categorical variables. Interobserver variability was assessed by selecting the T1 mapping sequences from all 30 patients with ARVC as well as a random sample of 30 first-degree relatives, which were then blindly reanalyzed by the senior investigator. Intraclass correlation coefficient (ICC) with 95% confidence interval was calculated to evaluate inter-operator reliability by using a two-way random-effects model. Intraclass correlation coefficient values > 0.75 were considered indicative of good reliability (and > 0.9 of excellent reliability) [21]. Statistical analysis was performed with STATA package, version 13.1 (StataCorp, College Station, Texas, USA). All statistical tests were two-tailed and a two-tailed value of P < 0.05 was considered significant throughout.

## Results

### Patients with ARVC

Characteristics of patients with ARVC are shown on Table 1. The mean age was 45 ± 27 years and 47% of the patients were male. Out of the 18 patients who underwent genetic testing, 9 (50%) carried a pathogenic or likely pathogenic variant in the PKP2 (n = 7, 39%) or the DSP (n = 2, 11%) genes. 18 Out of 30 patients had a positive family history for ARVC. The ECG was abnormal in 22 (73%) patients. The most common abnormalities were anterior TWI in V1–V3 in 16 patients (53%), lateral TWI in 4 patients (13%) and low QRS voltages in 4 (13%) patients (Table 1).

**Table 1:** Demographic characteristics and ECG indices in 30 patients with ARVC

Demographics	
Male, n (%)	14 (47)
Age (years)	45 ± 27
ECG	
SR, n (%)	28 (93)
HR, median (IQR) (bpm)	67 (55–75)
QRS duration, median (IQR) (ms)	86 (82–94)
QRS duration > 120 ms, n (%)	3 (10)
RBBB, n (%)	2 (6)
LBBB, n (%)	–
Low voltages precordial/limb leads^a^, n (%)	4 (13)
Q waves, n (%)	1 (3)
TWI V1–V3, n (%)	16 (53)
Lateral TWI, n (%)	4 (13)
Ventricular ectopic beats ≥ 1, n (%)	4(15)
Epsilon wave, n (%)	–

^a^QRS amplitude ≤ 1.0 mV in all of the precordial leads and/or QRS amplitude ≤ 0.5 mV in all of the limb leads

#### CMR in patients with ARVC

The CMR features of patients with ARVC are shown in Table 2. Cardiovascular magnetic resonance revealed structural or functional abnormalities in 26 (87%) patients. Patients (n = 4) with unremarkable CMR fulfilled TFC according to abnormalities in other cardiac tests.

**Table 2:** Main CMR features and T1 values in 30 patients with ARVC

CMR features	
LVEDV/BSA, median (IQR) (ml/m2)	79 (69–86)
LVEF, median (IQR) (%)	60 (57–64)
RVEDV, median (IQR) (ml)	178 (143–215)
RVEDV/BSA, median (IQR) (ml/m2)	94 (83–108)
RVESV/BSA, median (IQR) (ml/m2)	53 (44–59)
CMR major volume criteria^a^, n (%)	9 (30)
CMR minor volume criteria^a^, n (%)	2 (7)
RVEF, median (IQR) (%)	53 (44–59)
CMR major function criteria^a^, n (%)	4 (13)
CMR minor function criteria^a^, n (%)	3 (10)
RV RWMA, n (%)	20 (67)
	4 (13)
	11 (37)
	8 (27)
	5 (17)
LGE, n (%)	15 (50)
	4 (13)
	7 (23)
	4 (13)
	8(27)
	2 (7)
	2 (7)
	5 (17)
	7 (23)
	7 (23
LV involvement, n (%)	13 (43)

Native T1 mapping		
	1.5T (N = 18)	3T (N = 12)
IVS ROI area (cm^2^)	0.9 ± 0.3	1.2 ± 0.3
IVS mean value (ms)	977 ± 39 (n.v. 950 ± 21)	1189 ± 102 (n.v. 1052 ± 23)
Lateral ROI area (cm^2^)	0.8 ± 0.3	0.9 ± 0.3
Lateral mean value (ms)	970 ± 73	1129 ± 44
Abnormal IVS native T1, n (%)	3 (17)	8 (67)

*BSA* body surface area, *CMR* cardiovascular magnetic resonance, *IQR* inter-quartile range, *IVS* inter-ventricular septal, *LGE* late gadolinium enhancement, *LV* left ventricle, *LVEDV* left ventricular end-diastolic volume, *LVEF* left ventricular ejection fraction, *n.v.* normal values, *ROI* region of interest, *RV* right ventricle, *RVEDV* right ventricular end-diastolic volume, *RVEF* right ventricular ejection fraction, *RVESV* right ventricular end-systolic volume, *RVOT* right ventricular outflow tract, *RWMA* regional wall motion abnormalities, *SD* standard deviation

^a^According to the Revised Task Force Criteria for the diagnosis of ARVC

Isolated RV involvement was observed in 13 (43%) patients and isolated LV involvement in 2 (7%) patients. 11 (36 %) Patients exhibited biventricular abnormalities. The most common RV abnormality were RWMAs, observed in 20 (67%) patients (predominantly affecting the free wall and the right ventricular outflow tract in 11 and 8 patients, respectively) (Table 2). Right ventricular dilatation fulfilling a major or minor volume TFC was found in 11 (37%) patients and impaired RV systolic function (ejection fraction ≤ 45%) in 7 (23%) patients.

The main LV abnormality was myocardial LGE (n = 11; 36%), occurring mostly in the inferior or the lateral walls (8 out of 11 patients). A small proportion of patients exhibited LV RWMA (n = 2; 7%) or impaired (ejection fraction < 50%) systolic function (n = 2; 7%) (Table 2). In total, LGE was detected in 15 (50%) patients as follows: 4 (13%) with RV LGE only, 7 (23%) with isolated LV LGE and 4 (13%) patients with biventricular LGE distribution.

#### T1 mapping in patients with ARVC

**Fig. 1:**
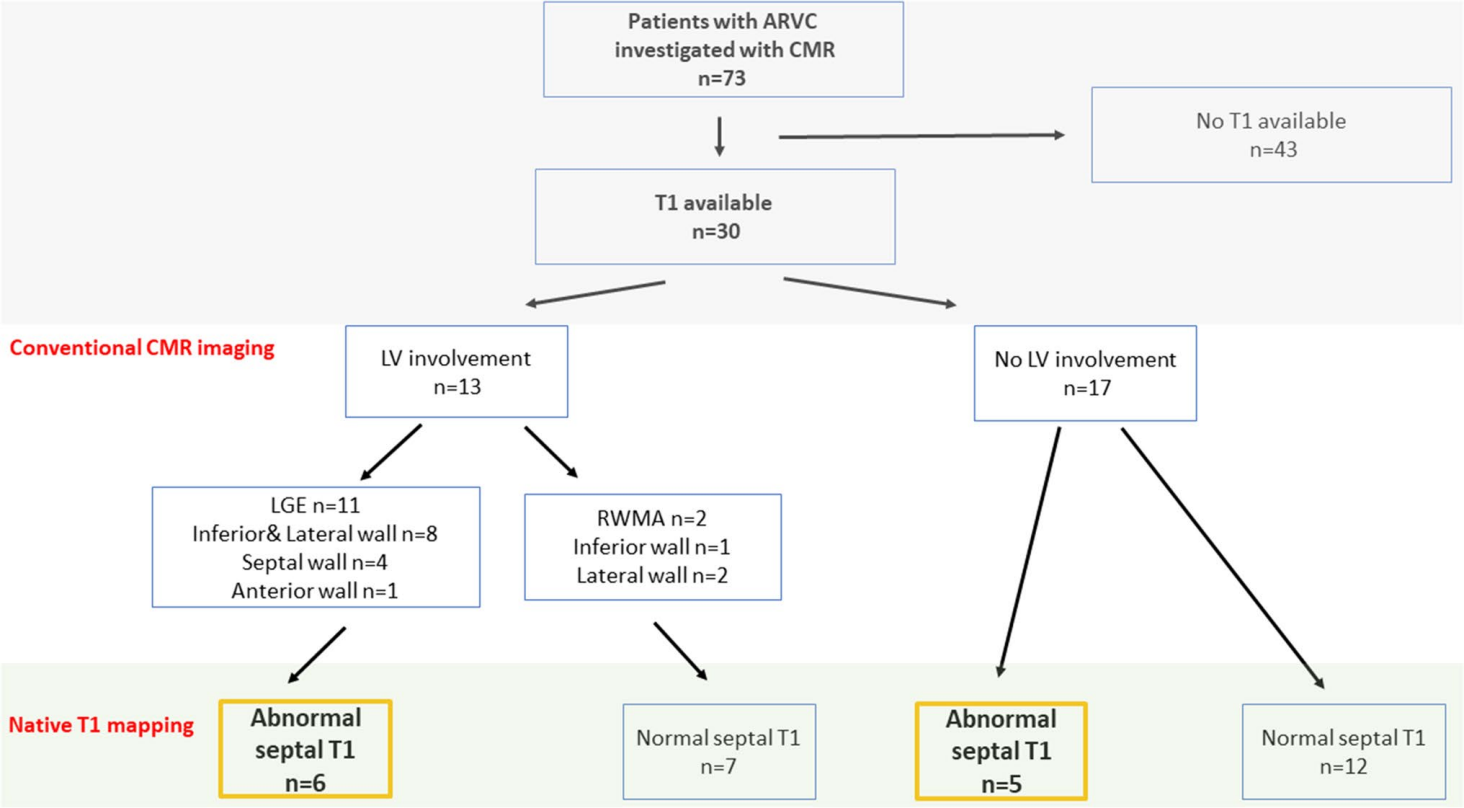
Flow chart and main characteristics of the patients with arrhythmogenic right ventricular cardiomyopathy (ARVC)

**Fig. 2:**
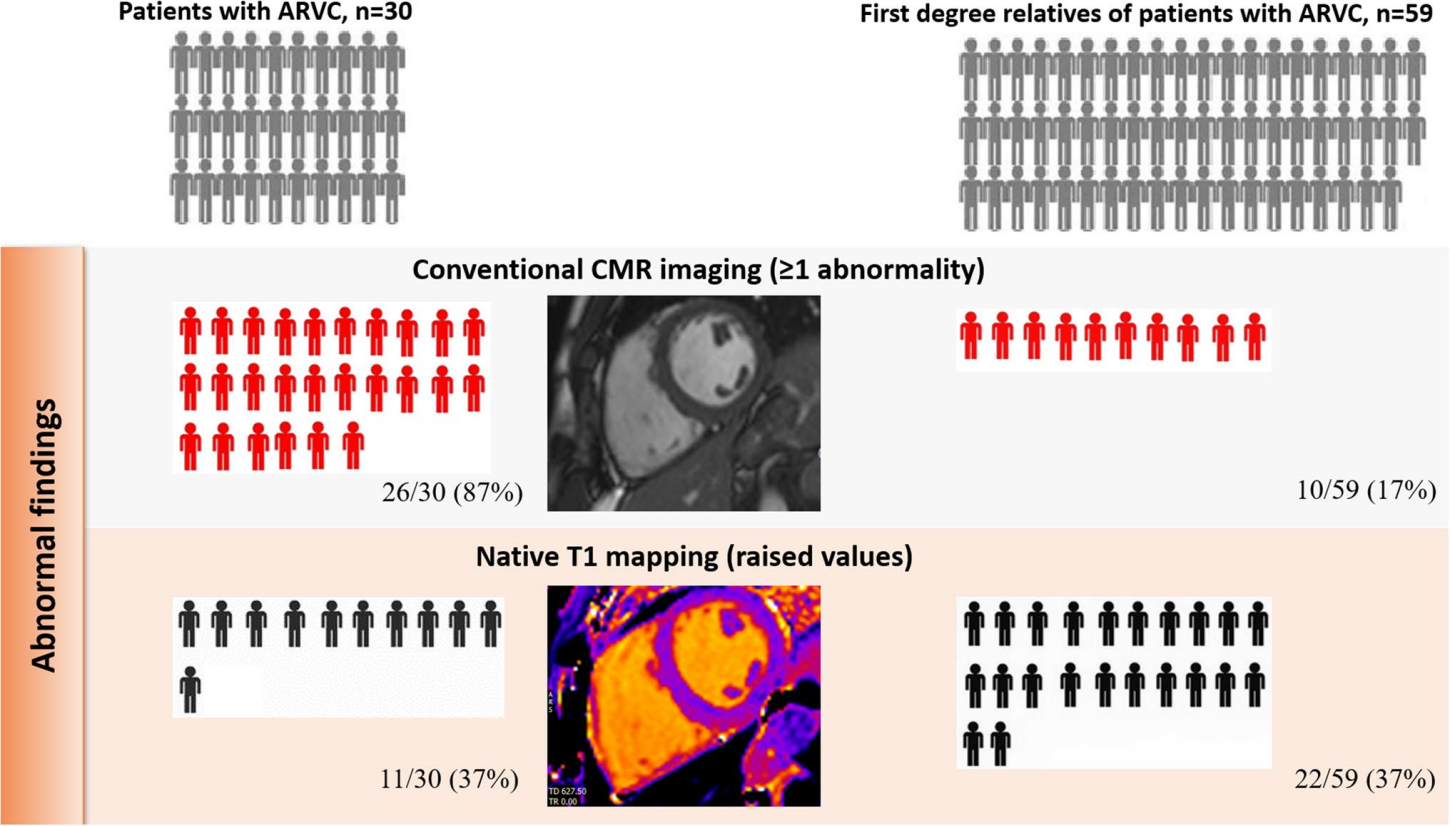
Abnormal findings by conventional cardiac magnetic resonance (CMR) imaging and native T1 mapping imaging

Native T1 values in patients with ARVC are shown in Table 2. According to pre-specified T1 mapping thresholds at the level of the interventricular septum (IVS), 11 (37%) of patients with ARVC revealed elevated values. No difference was observed in IVS native T1 values between patients with and without LV LGE (983 ± 14 (group with LGE) vs. 971 ± 11 (group without LGE), p = 0.515 and 1196 ± 45 (group with LGE) vs. 1185 ± 18 (group without LGE), p = 0.784 for 1.5T and 3T scanners, respectively). A similar proportion of patients with and without LGE exhibited elevated IVS native T1 values (50% vs. 28% respectively, p = 0.216). Overall, myocardial T1 values were abnormal in 5 out of the 17 patients who would have been classified as exhibiting a normal LV by conventional imaging (Figs. 1, 2).

No difference was observed with respect to ECG features between patients with normal and elevated myocardial T1 values (p > 0.05 for all) (Table 3).

**Table 3:** Differences in electrocardiographic features of patients with ARVC according to normal or abnormal myocardial T1 mapping

	Normal T1 (n = 19)	Abnormal T1 (n = 11)	P-value
TWI V1–V3, n (%)	10 (59)	6 (55)	0.823
Lateral TWI, n (%)	3 (16)	1 (9)	0.603
QRS duration > 120 ms, n (%)	1 (5)	1 (9)	0.685
Low voltages precordial/limb leads, n (%)	2 (12)	2 (20)	0.581
RBBB, n (%)	2 (12)	0 (0)	0.206
Ventricular ectopic beats ≥ 1	3 (16%)	1 (9%)	0.603
Ventricular tachycardia (LBBB pattern and superior axis)	7 (36.8%)	5 (45.5%)	0.656
Ventricular tachycardia (RVOT origin)	3 (15.8%)	2 (18.2%)	0.350

Data on ventricular tachycardia were based on ECG and ambulatory monitoring

*LBBB* left bundle branch block, *RBBB* right bundle branch block, *RVOT* right ventricle outflow tract, *TWI* T-wave inversion

### First‐degree relatives of patients with ARVC

Table 4 shows the main demographic characteristics and CMR parameters in 59 first-degree relatives who did not fulfil TFC diagnostic of ARVC. None of the family members were diagnosed with any other cardiac condition, apart from 4 individuals who exhibited features of hypertensive heart disease (i.e. increased LV mass or mild LV hypertrophy in the range of 12–15 mm). Genetic analysis (n = 16) revealed a pathogenic or likely pathogenic variant in the PKP2 (n = 7; 44%) and DSG (n = 1; 6%) genes.

**Table 4:** Demographic characteristics, CMR indices and myocardial T1 values in 59 first-degree relatives of patients with ARVC

Demographics	
Male, n (%)	24 (41)
Age, mean ± SD (years)	42 ± 19
Hypertensive heart disease, n (%)	4 (7)
CMR features	
LVEDV/BSA, median (IQR) (ml/m2)	77 (69–88)
LVEF, median (IQR) (%)	61 (59–64)
RVEDV, median (IQR) (ml)	145 (121–180)
RVEDV/BSA, median (IQR) (ml/m2)	77 (67–94)
RVESV/BSA, median (IQR) (ml/m2)	35 (26–41)
CMR major volume criteria, n (%)	–
CMR minor volume criteria, n (%)	2 (3)
RVEF, median (IQR) (%)	56 (53–61)
CMR major function criteria, n (%)	–
CMR minor function criteria, n (%)	1 (2)
RV RWMA, n (%)	8 (14)
RV apical RWMA, n (%)	1 (2)
RV free wall RWMA, n (%)	3 (5)
RV anterior wall/RVOT RWMA, n (%)	1 (2)
RV inferior RWMA, n (%)	3 (5)
LGE, n (%)	3 (5)
LGE LV only, n (%)	3 (5)
LGE infero-lateral, n (%)	3 (5)
LGE inferior, n (%)	3 (5)
LGE infero-lateral, n (%)	1 (2)
LGE inferior, n (%)	2 (3)

Native T1 mapping		
	1.5T (N = 42)	3T (N = 17)
IVS ROI area (cm^2^)	0.732 ± 0.204	0.808 ± 0.172
IVS mean value (ms)	992 ± 66.1 (n.v. 950 ± 21)	1155 ± 108 (n.v. 1052 ± 23)
Lateral ROI area (cm^2^)	0.779 ± 0.186	0.843 ± 0.215
Lateral mean value (ms)	997 ± 49	1122 ± 169
Abnormal IVS native T1, n (%)	10 (24)	12 (71)

**Fig. 3:**
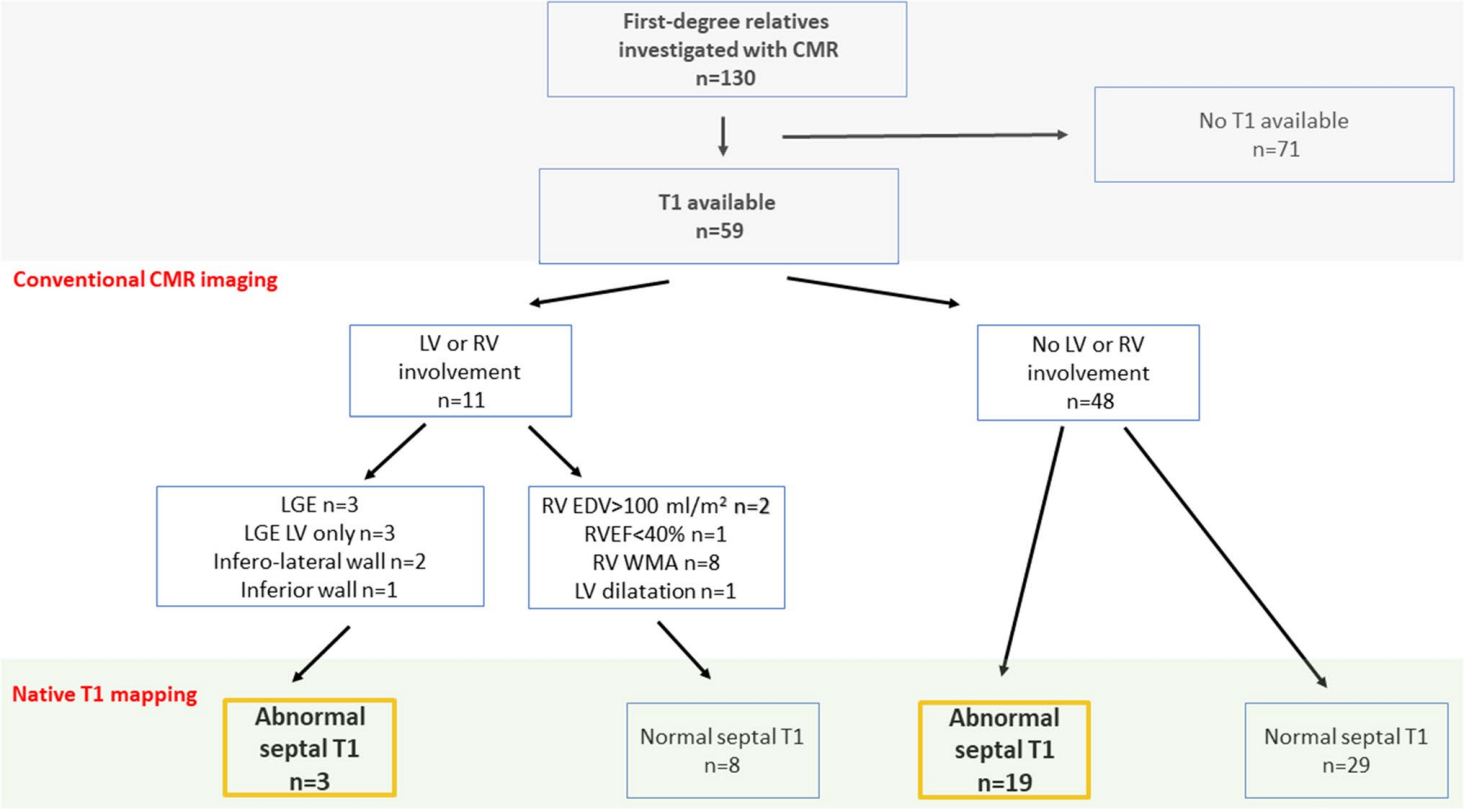
Flow chart and main characteristics of first-degree relatives of patients with ARVC

At least 1 CMR abnormality was found in 11 (19) first-degree relatives (Fig. 2). Isolated RV RWMAs were found in 8 (14) individuals and 1 individual fulfilled a single minor functional TFC (RVEF ≤ 45). Two individuals fulfilled minor volume TFC for ARVC (RV end-diastolic volume between 100 and 110 ml/m^2^). Late gadolinium enhancement was present in 3 (5) cases, predominantly the inferior wall (Table 4). In all the first-degree relatives exhibiting minor abnormalities at CMR, a comprehensive diagnostic work-up did not reveal any other feature suggestive of ARVC. Myocardial T1 values are shown in Table 4. 22 (37%) First-degree relatives exhibited elevated septal T1 values according to scanner specific thresholds (Fig. 3).

No association was observed between LV hypertrophy suggestive of hypertensive heart disease and abnormal T1 values (P = 0.106). Respectively, we did not find an association of genotype status and elevated T1 values (P = 0.999).

Measurements of average native T1 values showed good inter-observer reproducibility (ICC = 0.81, 95% CI 0.717–0.871).

## Discussion

Arrhythmogenic right ventricular cardiomyopathy is increasingly recognised as a biventricular disease [22, 23]. In this context, CMR is a powerful tool to detect structural and functional abnormalities, including RWMA, RV and/or LV systolic dysfunction and focal myocardial fibrosis through LGE imaging. Tissue characterization provides important clinical information beyond assessment of biventricular size and function. Our study shows that pre-contrast (or native) myocardial T1 values are often higher than normal in both patients with ARVC and first-degree relatives of patients with ARVC. Left ventricular involvement consisting of RWMA and LGE was observed in 43% of our patients with ARVC. Interestingly, native T1 mapping revealed elevated myocardial T1 values in a significant proportion of cases who would otherwise have been regarded as free of LV involvement by standard CMR techniques. In addition, over one-third (37%) of first-degree relatives not fulfilling current diagnostic criteria for ARVC revealed abnormal septal T1 values. No relationship was observed between potential LV involvement indicated by abnormal T1 values and ECG changes. It is possible that T1 changes are not reflected on the ECG because they represent a phase of very early LV involvement which does not find yet an electrical correlate.

### CMR features in ARVC

Structural RV changes based on CMR were incorporated into the revised diagnostic TFC published in 2010^2^. However, despite being initially considered a disease of the RV in isolation, recent studies have demonstrated that LV involvement is relatively common in ARVC [3, 24, 25].

Myocardial T1 mapping offers the opportunity to detect an increase in the extracellular space which may be due to diffuse fibrosis or myocardial infiltration [26]. Indeed, T1 mapping has been shown to be a useful technique in differentiating between specific cardiomyopathies characterized by left ventricular hypertrophy [27].

Our study shows that in addition to almost half of patients with ARVC exhibiting structural LV abnormalities and/or focal LV fibrosis, myocardial T1 values were abnormal in 5 of 17 patients in whom potential LV involvement would have otherwise remained undetected using conventional CMR imaging. In our cohort, we found that myocardial T1 was abnormal in 37% of patients, suggesting that T1 mapping may play a role in the detection of early or subtle LV involvement and may be complementary to other CMR sequences.

### CMR features in first‐degree relatives

A diagnosis of ARVC has significant implications for first-degree relatives. Since sudden cardiac death may be the first and only manifestation of disease, comprehensive evaluation of first-degree relatives is strongly recommended. The diagnostic work-up should include CMR, which can reveal abnormalities that may not be evident on other imaging techniques such as echocardiography.

Out of 59 first-degree relatives, 19% showed isolated CMR abnormalities which were not sufficient to provide a diagnosis of ARVC per se. Interestingly, 37% exhibited abnormally elevated septal T1 values. Although this finding suggests that CMR has the potential to detect early signs of LV disease in family members, results should be interpreted with caution and as merely descriptive. Several variables must be considered when T1 mapping analysis is performed including age, gender, comorbidities such as hypertension which may alter T1 values [27, 28]. Only 4 first-degree relatives showed features of mild hypertensive heart disease in our study and an association between increased T1 values and left ventricular hypertrophy was not observed.

The significance of abnormal septal T1 values in first-degree relatives remains uncertain. These findings will need to be corroborated by longitudinal studies aimed at demonstrating whether subtle changes revealed by T1 mapping predict the development of an overt phenotype in first-degree relatives at risk.

Our study has some limitations. This was a retrospective study and the sample size was relatively small. Although abnormal T1 values were derived from measurements at the level of the interventricular septum, we also analyzed T1 values at the level of the lateral LV wall where normal values have not yet been clearly established [29]. Finally, we used a ROI localized in the mid IVS and mid-lateral wall only, meaning that focal fibrosis or fat replacement elsewhere may have been missed. The choice of a specific ROI in the cohort studied was motivated by the need to have an analogous comparison with healthy individuals and therefore the methods used to assess normality in a previous study were followed [18].

## Conclusions

Patients with ARVC often exhibit LV involvement on CMR (43% of the cases in our study cohort). Native myocardial T1 values were higher than normal in 37% of patients, including a significant proportion of patients who would have been otherwise classified as exhibiting a normal LV using conventional CMR techniques. Abnormally elevated T1 values were also observed in more than one third of first-degree relatives who did not exhibit a cardiomyopathy phenotype after comprehensive investigations following a diagnosis of ARVC in their family members. The significance of abnormal septal T1 values in first-degree relatives remains uncertain and will require to be substantiated by future longitudinal studies.

## Supplementary Information

Below is the link to the electronic supplementary material.

(DOCX 21 kb)

## Data Availability

The datasets generated and/or analysed during the current study are not publicly available but are available from the corresponding author on reasonable request. Not applicable.

## Abbreviations

ARVC: Arrhythmogenic right ventricular cardiomyopathy
TFC: Task Force Criteria
LV: Left ventricular
CMR: Cardiovascular magnetic resonance
LGE: Late gadolinium enhancement
ECG: Electrocardiogram
TWI: T-wave inversion
LA: Left atrial
SSFP: Steady-state free precession
BSA: Body surface area
RWMA: Right ventricular regional wall motion abnormalities
ROI: Region of interest
