# Supplementary material for: Cardiac magnetic resonance in patients with ARVC and family members: the potential role of native T1 mapping
Source: Int J Cardiovasc Imaging. 2021 Feb 7;37(6):2037–47. doi: 10.1007/s10554-021-02166-7 (PMC8255264; doi:10.1007/s10554-021-02166-7)
Supplement: Supplementary file 1 — (DOCX 21 kb) [file 10554_2021_2166_MOESM1_ESM.docx]

**Supplemental material**

**Supplemental Table 1. The “Padua criteria” for the diagnosis of arrhythmogenic right ventricular cardiomyopathy.**

| **Category** | **RV** | **LV** |
| --- | --- | --- |
| I. Morpho-functional  ventricular  abnormalities | Echocardiography, CMR or angiography:  *Major*  • Regional RV akinesia, dyskinesia, or bulging plus one of the following:  - global RV dilatation (increase of RV EDV according to the imaging test specific nomograms)  - global RV systolic dysfunction (reduction of RV EF according to the imaging test specific nomograms)  *Minor*  • Regional RV akinesia, dyskinesia or aneurysm of RV free wall | Echocardiography, CMR or angiography:  *Minor*  • Global LV systolic dysfunction (depression of LV EF or reduction of echocardiographic global longitudinal strain), with or without LV dilatation (increase of LV EDV according to the imaging test specific nomograms for age, sex, and BSA)  *Minor*  • Regional LV hypokinesia or akinesia of LV free wall, septum, or both |
| II. Structural  myocardial  abnormalities | CMR:  *Major*  • Transmural LGE (stria pattern) of ≥1 RV region(s) (inlet, outlet, and apex in 2 orthogonal views)  EMB:  *Major*  • Fibrous replacement of the myocardium in ≥1 sample, with or without fatty tissue | CMR:  *Major*  • LV LGE (stria pattern) of ≥1 Bull's Eye segment(s) (in 2 orthogonal views) of the free wall (subepicardial or midmyocardial), septum, or both (excluding septal junctional LGE) |
| III. Repolarization  abnormalities | *Major*  • Inverted T waves in right precordial leads (V1, V2, and V3) or beyond in individuals with complete pubertal development (in the absence of complete RBBB)  *Minor*  • Inverted T waves in leads V1 and V2 in individuals with completed pubertal development (in the absence of complete RBBB)  • Inverted T waves in V1, V2, V3 and V4 in individuals with completed pubertal development in the presence of complete RBBB. | *Minor*  • Inverted T waves in left precordial leads (V4-V6) (in the absence of complete LBBB) |
| IV. Depolarization  abnormalities | *Minor*  • Epsilon wave (reproducible low-amplitude signals between end of QRS complex to onset of the T wave) in the right precordial leads (V1 to V3)  • Terminal activation duration of QRS ≥55 ms measured from the nadir of the S wave to the end of the QRS, including R', in V1, V2, or V3 (in the absence of complete RBBB) | *Minor*  • Low QRS voltages (<0.5 mV peak to peak) in limb leads (in the absence of obesity, emphysema, or pericardial effusion) |
| V. Ventricular  arrhythmias | *Major*  • Frequent ventricular extrasystoles (>500 per 24 h), non-sustained or sustained ventricular tachycardia of LBBB morphology  *Minor*  • Frequent ventricular extrasystoles (>500 per 24 h), non-sustained or sustained ventricular tachycardia of LBBB morphology with inferior axis (“RVOT pattern”) | *Minor*  • Frequent ventricular extrasystoles (>500 per 24 h), non-sustained or sustained ventricular tachycardia with a RBBB morphology (excluding the “fascicular pattern”) |
| VI. Family  history/genetics | *Major*  • ACM confirmed in a first-degree relative who meets diagnostic criteria  • ACM confirmed pathologically at autopsy or surgery in a first degree relative  • Identification of a pathogenic or likely pathogenetic ACM mutation in the patient under evaluation  *Minor*  • History of ACM in a first-degree relative in whom it is not possible or practical to determine whether the family member meets diagnostic criteria  • Premature sudden death (<35 years of age) due to suspected ACM in a first-degree relative  • ACM confirmed pathologically or by diagnostic criteria in a second-degree relative |  |

ACM, arrhythmogenic cardiomyopathy; BSA, body surface area; CMR, cardiovascular magnetic resonance; EDV, end-diastolic volume; EF, ejection fraction; LBBB, left bundle-branch block; LGE, late gadolinium enhancement; LV, left ventricle; RBBB, right bundle branch block; RV, right ventricle; RVOT, right ventricular outflow tract. Modified from: Corrado et al., Int J Cardiol. 2020.

**Supplemental Table 2. T1 mapping sequence for the 1.5T and 3.0T scanners.**

| **Scanners** | **3T scanner**  **Achieva TX,**  **Philips Healthcare,**  **the Netherlands** | **1.5T scanner**  **Ingenia,**  **Philips Healthcare,**  **the Netherlands** | **1.5 T scanner**  **Magnetom Aera,**  **Siemens Healthcare GmbH, Germany** |
| --- | --- | --- | --- |
| **Coil** | 32-channel | | |
| **Technique** | T1 mapping | | |
| **Sequence** | 2D Breath-hold MOdified Look-Locker Inversion-Recovery (MOLLI) | | |
| **Parameters** | Acquisition Sampling Scheme:  a) pre-contrast (native): 5(3)3  b) Post-contrast: 4(1)3(1)2  Read-out: single-shot SSFP with trigger delay in mid-diastole.  Flip angle: 50°,  field of view: 320 × 320,  TR/TE: 330/1.57,  interpolated voxel size: 0.9 × 0.9 × 8 mm,  166 phase-encoding steps,  heart rate–adapted trigger delay,  11 phases (3 + 3 + 5), and adiabatic pre-pulse to achieve a complete inversion | | Acquisition Sampling Scheme:  a) pre-contrast (native): 5(3)3  b) Post-contrast: 4(1)3(1)2  Read-out: single-shot SSFP with trigger delay in mid-diastole.  Flip angle: 35°; TR/TE: 272/1.2 ms; BW: 1085 Hz/Pixel; voxel size: 1.5x1.5x8mm3; PAT: 2; number of inversion pulse (180°) 2 and 3 for native and post-contrast, respectively |
| **Notes on Image Generation** | Motion correction image preparation step using a custom-made tool developed in house on the basis of a hierarchical adaptive local affine registration technique, Co-registered images were then used to derive T1 values. | | Generation of inline motion corrected pixel-based T1-maps by acquiring a series of images over several heartbeats with shifted T1 times corrected by RR duration |
